# Vitamin D and activated vitamin D in tuberculosis in equatorial Malaysia: a prospective clinical study

**DOI:** 10.1186/s12879-017-2314-z

**Published:** 2017-04-27

**Authors:** Anna P. Ralph, Muhammad Redzwan S. Rashid Ali, Timothy William, Kim Piera, Uma Parameswaran, Elspeth Bird, Christopher S. Wilkes, Wai Khew Lee, Tsin Wen Yeo, Nicholas M. Anstey

**Affiliations:** 1 Global and Tropical Health Division, Menzies School of Health Research, Charles Darwin University, PO Box 41096, Casuarina, NT 0811 Australia; 2 Department of Medicine, Royal Darwin Hospital, Darwin, Northern Territory Australia; 3 Infectious Diseases Society Sabah-Menzies School of Health Research Clinical Research Unit, Kota Kinabalu, Sabah Malaysia; 4 Department of Respiratory Medicine, Queen Elizabeth Hospital, Kota Kinabalu, Sabah Malaysia; 5 Jesselton Medical Centre, Kota Kinabalu, Sabah Infectious Diseases Unit, Clinical Research Centre, Queen Elizabeth Hospital, Kota Kinabalu, Sabah Malaysia; 6 Clinical Research Centre, Queen Elizabeth Hospital, Kota Kinabalu, Sabah Malaysia; 7 Luyang Outpatient Clinic, Kota Kinabalu, Sabah Malaysia; 8 Lee Kong Chian School of Medicine, Nanyang Technological University, Singapore, Singapore

**Keywords:** 25-hydroxyvitamin D, Cholecalciferol, 1,25-dihydroxyvitamin D, Calcitriol, Tuberculosis

## Abstract

**Background:**

Vitamin D deficiency (low plasma 25-hydroxyvitamin D [25D] concentration) is often reported in tuberculosis. Adjunctive vitamin D has been tested for its potential to improve treatment outcomes, but has proven largely ineffective. To better understand vitamin D in tuberculosis, we investigated determinants of 25D and its immunologically active form, 1,25-dihydroxyvitamin D (1,25D), their inter-relationship in tuberculosis, longitudinal changes and association with outcome.

**Methods:**

In a prospective observational study of adults with smear-positive pulmonary tuberculosis in Sabah, Malaysia, we measured serial 25D, 1,25D, vitamin D-binding protein (VDBP), albumin, calcium, parathyroid hormone, chest x-ray, week 8 sputum smear/culture and end-of-treatment outcome. Healthy control subjects were enrolled for comparison.

**Results:**

1,25D was elevated in 172 adults with tuberculosis (mean 229.0 pmol/L, 95% confidence interval: 215.4 - 242.6) compared with 95 controls (153.9, 138.4-169.4, *p* < 0.001), directly proportional to radiological severity (*p* < 0.001), and fell rapidly within one week of treatment commencement. Tuberculosis patients with higher baseline 1,25D achieved significantly higher percentage weight gain over time, including when controlling for baseline weight, however persistently elevated 1,25D was associated with worse residual x-ray changes and lower end-of-treatment BMI. 1,25D was inversely associated with PTH (*p* < 0.001), consistent with the extra-renal origin of the 1,25D. 25D did not differ between tuberculosis patients (mean 63.9 nmol/L, 95% CI: 60.6 - 67.3) and controls (61.3, 57.2- 65.3, *p* = 0.24), and was unassociated with outcomes. Among tuberculosis patients in multivariable analyses, sex, age and VDBP were associated with 25D, and age and albumin with 1,25D. 1,25-dihydroxyvitamin was not significantly asscociated with 25D. Vitamin D deficiency <25 nmol/L was uncommon, occurring in only five TB patients; 1,25D was elevated in three of them.

**Conclusions:**

In an equatorial setting, high extra-renal production of 1,25D was seen in tuberculosis, including in individuals with 25D in the deficient range; however, severe 25D deficiency was uncommon. Baseline elevation of 1,25D, a marker of macrophage activation, was associated with better weight gain but persistent elevation of 1,25D was associated with worse radiological and BMI outcomes. 1,25D warrants testing in larger datasets including TB patients less responsive to treatment, such as multi-drug resistant TB, to test its utility as a marker of tuberculosis severity and treatment response.

## Background

Tuberculosis (TB) remains a leading global cause of morbidity and mortality [1]. Vitamin D metabolism is altered in tuberculosis [2, 3], and a number of studies have described vitamin D deficiency in active tuberculosis [3–5]. However, more recent studies from equatorial African settings have provided divergent results [6, 7]. Thus it appears that while profound vitamin D deficiency may pose a risk for the development of active TB, this effect is not observable in equatorial locations which are relatively protected from vitamin D deficiency.

Vitamin D deficiency is defined according to serum concentration of total (free and protein-bound) 25-hydroxyvitamin D_3_ (25D, also termed cholecalciferol). Deficiency is common currently, partly because the thresholds cited for deficiency are not based on normal population distributions, and are now lower than in previous decades, as reviewed elsewhere [8]. The active form of vitamin D is 1,25-dihydroxycholecalciferol (1,25D, also termed calcitriol).1,25D can be produced in excess in tuberculosis occasionally resulting in hypercalcaemia [9–11], due to production of 1-α hydroxylase by activated macrophages [12] in an unregulated fashion [13]. Activated vitamin D has key immunomodulatory functions in the human anti-mycobacterial response [8, 12, 14, 15], so the ability of infected human host cells to generate and utilise adequate intracellular 1,25D is considered to be of major importance. Increased production of 1,25D in TB is integral to expression of an anti-mycobacterial peptide, stimulation of autophagy and autophagosome activity [14] and reduction in deleterious cell-mediated inflammatory responses [16].

These factors and other apparent associations between vitamin D and TB (e.g. seasonality [17]), have fuelled optimism that vitamin D adjunctive treatment may be beneficial in preventing or treating TB [16, 18, 19]. However, findings from trials in active TB have mostly been negative to date [20–24]. Alternative hypotheses may explain the apparent associations; e.g. vitamin D ‘deficiency’ could be a consequence of tuberculosis, since vitamin D behaves as a negative acute phase reactant in other situations such as surgery [25–27] and during immune restoration syndrome in TB-HIV co-infection [28].

No studies in which 25D and 1,25D have both been measured in TB have been reported to date. While 1,25D is the active hormone, its measurement has generally not been performed because of its reported short half-life, 1000-fold lower concentration than 25D, because levels may rise in vitamin D deficiency in response to elevated PTH [29] and because systemic levels might not reflect true concentration at the intracellular level [30]. Also, *in vitro* studies show that immunological effects may correlate better with 25D concentrations [13]. Nevertheless, measurement of both serum 25D and 1,25D would provide answers about the origins and consequences of low 25D in active TB. Speculation to date has included that 25D deficiency would result in 1,25D concentrations insufficient for optimal immunological responses [14], or that 25D deficiency may occur due to excessive conversion to 1,25D [31].

Our aims were to identify determinants of 25D and 1,25D concentrations in active tuberculosis, examine longitudinal changes in the absence of vitamin D supplementation, and explore relationships between 25D and 1,25D and with disease severity and TB outcomes. We therefore conducted a prospective study of adults with pulmonary TB in Sabah, Malaysia. We also recruited healthy volunteers for comparison.

## Methods

### Setting

This was a prospective observational study of smear-positive pulmonary tuberculosis (PTB) outpatients in Sabah, Malaysia. Kota Kinabalu is equatorial, latitude 6°N, with fairly consistent weather year round. Participants were enrolled at Luyang Tuberculosis Outpatient Clinic, Kota Kinabalu.

### Ethics, consent and permissions

Eligible participants with PTB and healthy controls were required to provide written, informed consent after receiving verbal, written and pictorial explanations about the study. Consent was obtained from a parent/guardian for participants aged 15–17. Ethical approval was obtained from the Medical Research Ethics Committee, Malaysian Ministry of Health (NMRR-11-1051-10491), and the Human Research Ethics Committee of the Northern Territory Department of Health and Menzies School of Health Research, Australia (HREC-2010-1398).

### Participants

Participants were eligible if they provided consent, had sputum smear-positive pulmonary TB, were aged ≥15 years, not pregnant, and had received <7 days’ TB treatment. Smear-positive pulmonary TB was diagnosed on the basis of clinical and x-ray assessment, and at least one sputum positive for acid fast bacilli (AFB) on Ziehl-Neelsen stain. Culture confirmation of *Mycobacterium tuberculosis* was obtained [32]; patients in whom TB was confidently excluded (e.g. non-tuberculous mycobacteria or negative culture with inadequate clinical evidence of TB) were excluded from analyses. Healthy controls, sought from among family members of TB patients, blood donors, and clinic staff members, were eligible to volunteer if they provided consent, were aged ≥18 years, not pregnant, and had no intercurrent illness including cough or asthma. Any TB contacts had mantoux testing performed followed by chest x-ray if positive, and exclusion as a healthy control if TB or other disease was identified. Participants lost to study follow up were re-engaged whenever possible if they attended the TB clinic to collect medication or for clinical review. For participants who switched treatment clinics, multiple contact attempts were made by telephone and text message to the individual or their next of kin to ask them to resume study participation at Luyang clinic and to ensure they were adhering to TB treatment; participants who defaulted from TB treatment altogether were similarly contacted multiple times by study staff, in addition to activation of the routine defaulter tracing service provided by Luyang clinic.

### Procedures

Blood was collected for 25D, 1,25D, parathyroid hormone (PTH), calcium, albumin and vitamin D binding protein once in controls and at serial time points in tuberculosis patients (weeks 0, 1, 8, and 24; week 1 blood samples were introduced later and hence have more missing values). HIV was additionally tested in TB patients [33]. After collection, blood was separated immediately at the on-site laboratory, by centrifugation at 2000 rpm for 6 min. Aliquots were promptly frozen at −80 °C and batched for transportation to the reference laboratories at regular intervals. Assays used were: 25-OH vitamin D_2_ and 25-OH vitamin D_3_: liquid chromatography-tandem mass spectrometry; 1,25 dihydroxyvitamin D: IDS-iSYS immunopurification and quantitative determination; vitamin D-binding protein: Immunodiagnostik Enzyme linked immunosorbent assay; PTH: Roche cobas electrochemiluminescence immunoassay; calcium and albumin: Abbott ARCHITECT c16000 analyzer (Abbott Diagnostics, Chicago, IL, USA). Chest x-rays were taken in TB patients at enrolment, 8 weeks and 24 weeks, and read by a single investigator (APR) blinded to blood results using a previously validated score [34].

### Definitions

Ethnicity was described as Malaysian (Malaysian-born people of Malay, Chinese and/or Indian descent), and non-Malaysian (Indonesian or Filipino). The threshold for adequate (sufficient) 25D was considered to be ≥50 nmol/L [35]. Normal reference ranges for other assays provided by the respective laboratories were: 1,25D: 50–160 pmol/L, PTH: 0.8-5.5 pmol/L, corrected calcium: 2.10-2.55 mmol/L, albumin: 39–50 g/L.

### Statistics

Data were analysed using Stata^TM^ 14.0. (Stata Corp, College Station, Texas, USA). No sample size calculation was required for this observational study design. Continuous variables were compared using Student’s T test or Wilcoxon Rank Sum test. Normal transformations were applied where necessary (PTH: log; VDBP: square-root). Categorical variables were compared using Chi-squared or Fisher’s Exact test, as appropriate. Multivariable linear or logistic regression models were developed using a forwards stepwise approach; variables showing potential association with the dependent variable or those considered plausibly relevant (e.g. age, sex, body mass index [BMI]), were included in final models. The goodness of fit of logistic regression models was assessed using the Hosmer-Lemeshow test. For linear regression models, residuals were examined to ensure that relevant assumptions were met. For longitudinal data, analyses were restricted to individuals with paired data (baseline result and at the follow up time point), and compared using paired t-tests.

## Results

**Fig. 1:**
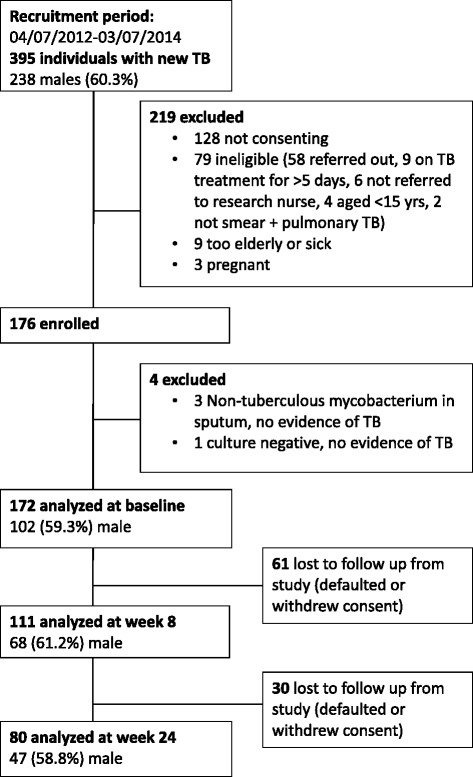
Study diagram

After exclusions (Fig. 1), 172 sequential, consenting participants were included for analysis. Most (148; 84.1%) had received ≤2 doses of TB treatment prior to enrolment and none had received >7 doses. Other results from this patient cohort have been published showing none had multidrug-resistant TB [32] and 3 (1.7%) had HIV co-infection [33]. Men were over-represented, were more likely to smoke, had a tendency toward higher baseline smear grade, and had more extensive radiological involvement (Table 1). Greater disease severity in men remained evident after controlling for smoking (data not shown). Ninety six healthy controls were enrolled; they were more likely to be Malaysian, female, non-smokers and have higher BMI than TB patients (*p* < 0.0001 in each instance); see Table 1. These factors were controlled for in multivariable analyses.

**Table 1:** Baseline characteristics

	Tuberculosis	Controls
Number	172	95
Age in years: median (range)	29 (15–70)	28 (18–66)
Male: no. (%)	102 (59.3%)	34 (35.8%)
Malaysian: no. (%)	113/172 (65.7%)	86/95 (90.5%)
Current or ex-smoker	88/172 (51.2%)	27/95 (28.4%)
Males	80/102 (78.4%)	23/34 (67.7%)
Females	8/70 (11.4%),	4/61 (6.6%)
Body mass index (kg/m^2^): mean (range)	18.0 (9.95-31.1)	24.2 (16.1-37.2)
HIV positive: no. (%)	3/172 (1.7%)	-
Microscopy grade ≥2 plus: no. (%)	118/175 (67.4%)	-
Males	75/103 (72.8%)	
Females	43/72 (59.7%)	
Time to culture positivity in days: mean (standard deviation)	13.5 (3.9)	-
Radiological severity:		
Cavitary disease	94/144 (65.2%)	-
Score: mean (standard deviation)	69.9 (34.5)	
Males	74.8 (33.7)	
Females	61.4 (35.4)	

The median time of freezing of blood samples after collection was 15.0 min, with 275/320 (85.9%) samples being frozen within 30 min. Results were not available for all assays at all time-points, due to failures of patients to attend appointments, inadequate aliquots for all assays, or laboratory errors. Losses to follow up are shown in Fig. 1.

### Tuberculosis versus controls

**Fig. 2:**
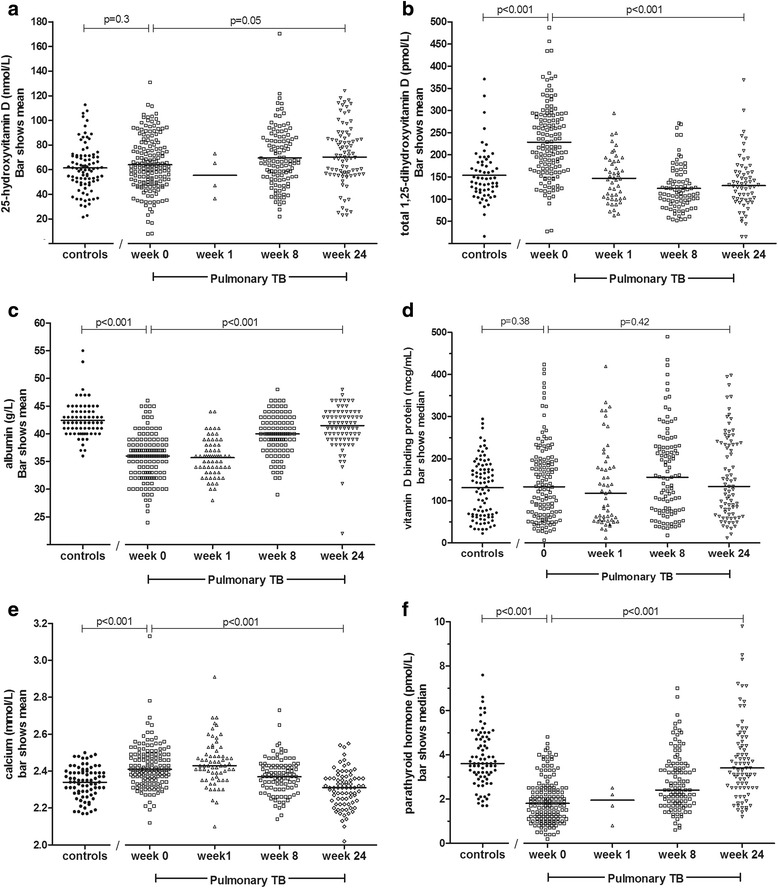
Blood test results at baseline and during follow up in TB patients and healthy controls. Legend: An individual data point is shown for each available test result. Longitudinal statistical comparisons of results are restricted to individuals with paired data. (**a**) 25-hydroxyvitamin D (**b**)1,25-dihydroxyvitamin D (**c**) albumin (**d**) vitamin D binding protein (**e**) calcium (**f**) parathyroid hormone

**Fig. 3:**
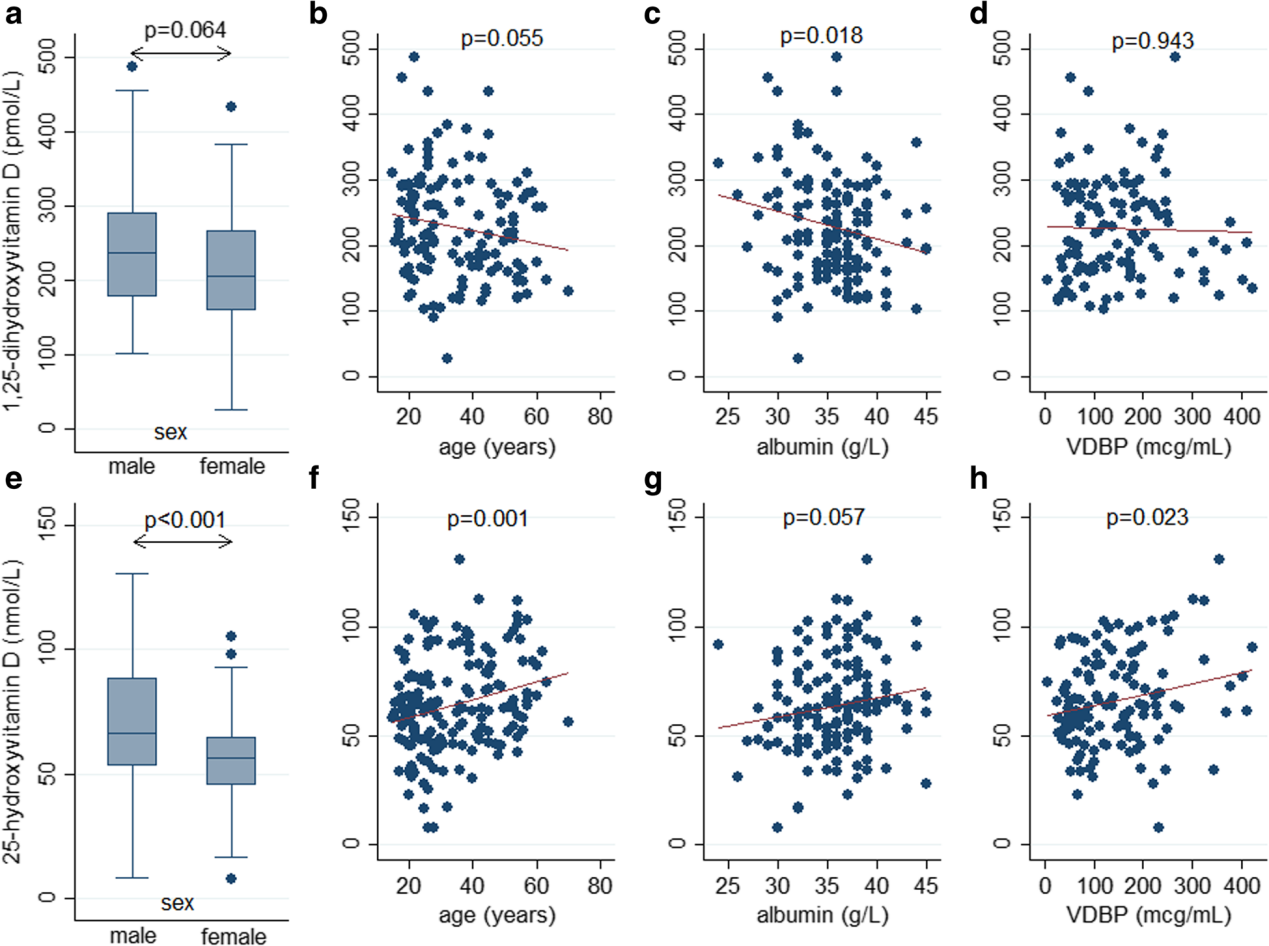
Associations of sex, age, albumin and vitamin D binding protein with 1,25-dihydroxyvitamin D and 25-dihydroxyvitamin D among tuberculosis patients at baseline. (**a**) 1,25-dihydroxyvitamin D by sex (**b**) 1,25-dihydroxyvitamin D by age (**c**) 1,25-dihydroxyvitamin D by albumin (**d**) 1,25-dihydroxyvitamin D by vitamin D binding protein (**e**) 25-hydroxyvitamin D by sex (**f**) 25-hydroxyvitamin D by age (**g**) 25-hydroxyvitamin D by albumin (**h**) 25-hydroxyvitamin D by vitamin D binding protein

At enrolment, mean total 1,25-dihydroxyvitamin D was elevated in tuberculosis (mean 229.0 pmol/L, 95%CI 215.4-242.6) compared with controls (153.9, 138.4-169.4, *p* < 0.001). Calcium was also significantly higher, PTH and albumin were significantly lower, and VDBP did not differ (Table 2; Fig. 2). Mean total 25D did not differ between TB patients and healthy controls in univariable analysis nor when controlling for sex, age, BMI and ethnicity (Table 2). Among both TB patients and controls, males had significantly higher 25D than females (Table 2 and Fig. 3e).

**Table 2:** Blood test results among tuberculosis patients at diagnosis and controls

	Tuberculosis	Controls	Univariable analyses, TB vs controls		Multivariable analyses, TB vs controls	
			β coefficient	*P* value	β coefficient	*P* value
Total 25-hydroxyvitamin D (nmol/L): mean (95% CI)						
All	63.9 (60.6- 67.3)	61.4 (57.4- 65.3)	2.55	0.350	−4.11	0.238
Males	69.9 (65.5-74.3)	70.1 (64.4-75.9)				
Females	55.2 (50.6-59.8)	56.2 (51.4-61.0)				
Vitamin D deficiency (25D < 50 nmol/L)	46/167 (27.5%)	23/92 (25.0%)		0.657		
Total 1,25-dihydroxyvitamin D (pmol/): mean (95% CI)	229.0 (215.4-242.6)	153.8 (138.6-169.0)	75.20	<0.001	63.5	<0.001
Parathyroid hormone (pmol/L): median (IQR)	1.8 (1.1-2.5)	3.6 (3.0-4.7)	−0.76	<0.001	−0.71	<0.001
Calcium: mean (95% CI)	2.42 (2.40-2.44)	2.34 (2.32-2.36)	0.08	<0.001	0.09	<0.001
Albumin: mean (95% CI)	35.7 (35.1-36.3)	42.4 (41.7-43.1)	−6.73	<0.001	−5.64	<0.001
Vitamin D binding protein (mcg/mL): median (IQR)	128.0 (IQR 66.6-198.3)	131.6 (66.0-178.4)	16.30	0.301	5.06	0.818

All models include age, sex, ethnicity, BMI. Transformations were applied to achieve a normal distribution for PTH (log) and VDBP (square root)

### Factors associated with 1,25-dihydroxyvitamin D and 25-hydroxyvitamin D in TB

**Fig. 4:**
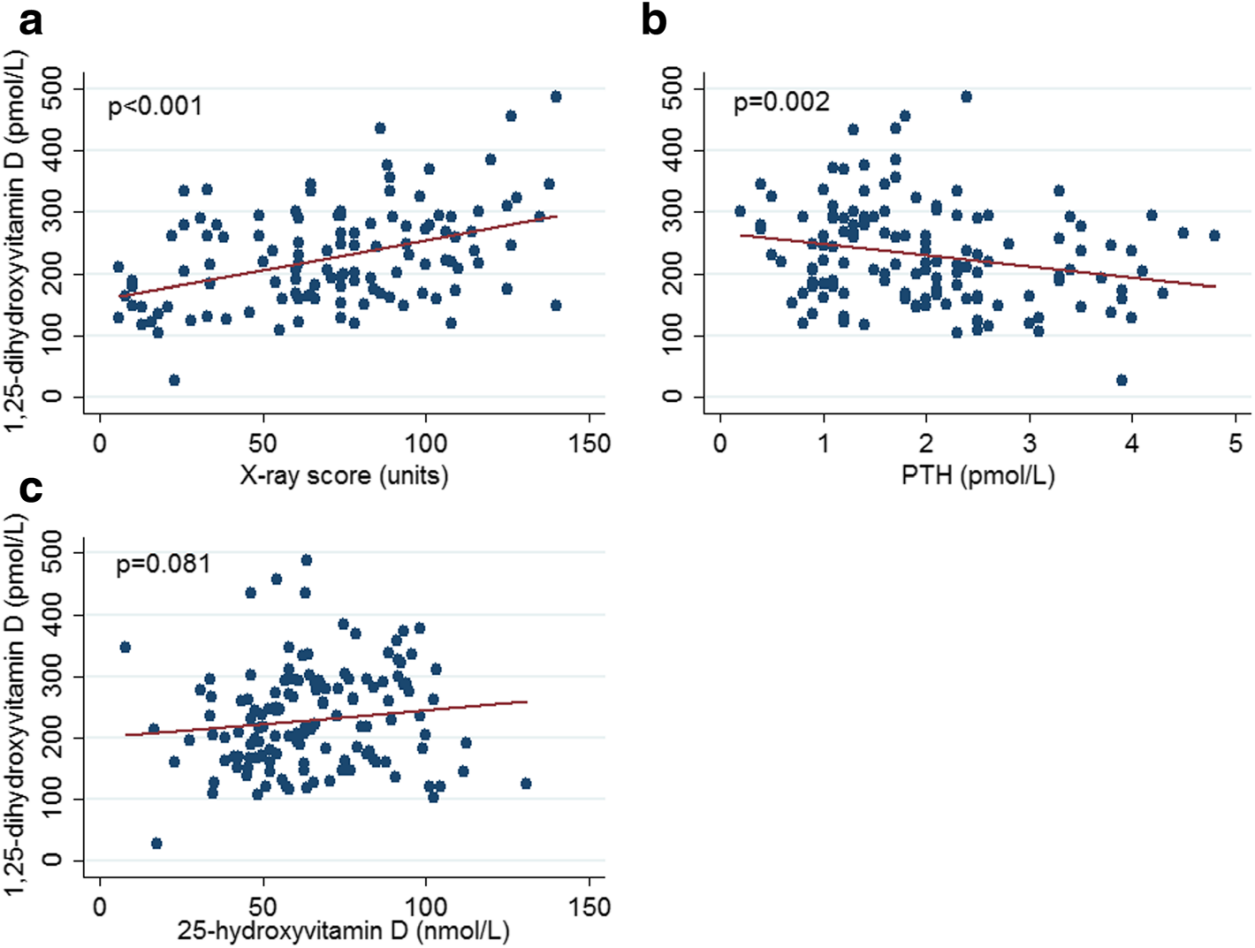
Relationships between 1,25-dihydoxyvitamin D and radiological severity, parathyroid hormone and 25-hydroxyvitamin D at baseline. (**a**) 1,25-dihydroxyvitamin D by x-ray score (**b**) 1,25-dihydroxyvitamin D by parathyroid hormone (**c**) 1,25-dihydroxyvitamin D by 25-hydroxyvitamin D

Factors associated with 1,25D and 25D are shown in Table 3 and Figs. 3 and 4. 1,25D was strongly associated with radiological severity (Fig. 4a), and inversely associated with PTH (Fig. 4b). There was no statistically significant relationship between 25D and 1,25D (*p* = 0.08; Fig. 4c). Mean 1,25D concentration was lower (median 159 pmol/L, range 27–346) in those with severe vitamin D deficiency <25 nmol/L compared to those with a level >25 nmol/L (median 218 pg/mL, range 106–456), but this was not statistically significant *p* = 0.16. Severe vitamin D deficiency did not preclude a high 1,25D concentration; for example, one male with severe vitamin D deficiency (serum 25D concentration 8.0 nmol/L) had a corresponding 1,25D concentration of 346 pmol/L (Fig. 4c).

**Table 3:** Factors associated with 1,25-hydroxyvitamin D and 25-hydroxyvitamin D in tuberculosis patients and control subjects

	25-hydroxyvitamin D				1,25-hydroxyvitamin D			
	Univariable analyses		Multivariable analyses		Univariable analyses		Multivariable analyses	
	β coefficient	*P* value	β coefficient	*P* value	β coefficient	*P* value	β coefficient	*P* value
Tuberculosis								
Sex (0 = female, 1 = male)	14.65	**<0.001**	−12.80	**0.007**	25.85	0.064	−34.37	0.057
Age	0.41	**0.001**	0.32	**0.024**	−0.98	0.055	−1.83	**0.001**
BMI	0.31	0.561	-	-	−3.59	0.111	-	-
Smoking (0 = no, 1 = yes)	12.91	**<0.001**	4.84	0.062	5.13	0.711	−7.66	0.468
Albumin	0.86	0.057	0.46	0.356	−4.14	**0.018**	−4.52	**0.021**
VDBP	0.04	**0.023**	**0.03**	**0.037**	0.00	0.943	0.00	0.514
X-ray severity score	−0.09	0.125	−0.11	0.054	0.97	**<0.001**	0.68	**0.003**
Controls								
Sex (0 = female, 1 = male)	13.92	**0.001**	−8.64	0.077	1.00	0.744	−35.40	0.23
Age	0.42	**0.012**	0.04	**0.045**	−1.14	0.062	−1.69	0.08
BMI	−0.21	0.637	-	-	−1.40	0.453	-	-
Smoking (0 = no, 1 = yes)	12.15	**0.009**	3.19	0.471	1.00	0.497	2.40	0.92
Albumin	0.57	0.424	0.13	0.858	0.55	0.885	−3.92	0.35
VDBP	0.03	0.175	0.00	0.778	0.07	0.389	0.00	0.17

*P* values ≤0.05 shown in bold

Multivariable models include sex, age, smoking, albumin, VDBP and for TB patients, x-ray score

A transformation was applied to achieve a normal distribution for VDBP (square root)

### Longitudinal changes

Among tuberculosis patients, 1,25D decreased rapidly after institution of treatment, reaching the normal reference range by week 1 (Fig. 2b). Albumin rose significantly over time, and VDBP concentration did not change (Fig. 2c and d). A small increase in total 25D occurred among 81 individuals with paired data during treatment (Fig. 2a); 25D increased from 68.0 at week 0 to 71.7 at week 24, *p* = 0.049.

### Associations with microbiological and clinical outcomes

**Fig. 5:**
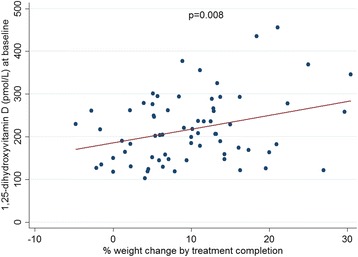
Relationship between baseline 1,25-dihydroxyvitamin D and percentage weight gain during tuberculosis treatment

TB treatment outcomes were recorded for 159 individuals. One death and no failures were reported (cured/completed: 112 (70.4%), defaulted: 23 (14.5%), transferred 23 (14.5%), died 1 (0.6%)). At 2 months, 8 were reported culture positive and 11 were smear positive. The small numbers limited the ability to explore associations; no associations with 2-month culture conversion were identified. In testing associations with other outcome measures (percentage lung affected on x-ray, overall x-ray score and BMI at treatment completion), 25D was unassociated with treatment outcome. However, 1,25D did show associations with x-ray and BMI outcomes. Individuals with higher baseline 1,25D achieved significantly higher percentage weight gain over time (Fig. 5), including when controlling for baseline weight (itself directly proportional to %weight gain); weight gain by treatment completion was 3.2% greater for every 1pmol/L increase in baseline 1,25D (*p* = 0.008). A similar statistically significant association was found for BMI. A failure of 1,25D to normalise to less than 150 pmol/L after week 0 was associated with poorer final radiological outcome (Table 4). Persistent elevation in 1,25D at week 24 was also associated with poorer weight gain (BMI 18.2 versus 20.4 kg/m2, *p* = 0.008). These associations were not explained by a relationship between baseline 1,25D and baseline or final disease severity (Fig. 4a), since elevation in 1,25D >150 pmol/L at baseline was not associated with these measures.

**Table 4:** Relationship between a persistent elevation in 1,25D and radiological severity at treatment completion

	1,25D ≤150 pmol/L	1,25D >150 pmol/L	
	X-ray score at treatment completion (median, range)		*P* value
week 0	10 (1–101)	17 (0–115)	0.460
week 1	8.5 (2–68)	33.5 (1–83)	0.075
week 8	12.0 (0–101)	36.5 (5–115)	0.033
week 24	11.0 (0–101)	42.0 (2–115)	0.002
Pooled results (any week after enrolment)	11.0 (0–101)	42 (1–115)	<0.001

Results show that 1,25D >150 pmol/L at week 0 was unassociated with final radiological outcome, but persistent elevation in 1,25D >150 pmol/L at weeks 1, 8 or 24 was associated with worse final radiological outcome, i.e. a higher x-ray score

## Discussion

Among people with active pulmonary TB, we found that systemic concentrations of total activated vitamin D (1,25D) were very high, in direct proportion to disease severity, fell rapidly with institution of effective therapy, and were more likely to remain elevated above 150 pmol/L in individuals with persisting radiological changes and poorer BMI attainment. High 1,25D was associated with suppressed PTH responses as expected, in keeping with macrophage-derived 1α-hydroxylase as the source of the elevated 1,25D. We cannot exclude that the sudden fall in 1,25D with treatment was due to a drug effect; isoniazid and rifampicin can both affect 25D concentration [36] although an effect on 1,25D has not been reported. However, the associations with outcomes suggest that even if this were a factor, it is not the sole explanation.

Vitamin D deficiency was not a characteristic of TB in this tropical setting, and was unassociated with TB outcomes. A similar lack of association has also been shown in a study of Malawian adults with TB [37]. 1,25D was not closely linked to serum 25D concentration.

These findings contrast with some previous results from non-tropical settings, where 25D concentration is frequently lower in active TB then controls [3–5] and where there may be failure to mount an adequate 1,25D response [38]. Our findings support the hypothesis that individuals who have adequate vitamin D stores at the time of onset of active TB are able to produce 1,25D from extra-renal sources in excess. It is plausible that a threshold effect may occur whereby serum 25D concentrations in the very deficient range, of which we saw little, would indeed not support the high 1,25D production shown in our patients. This could potentially explain differences in findings reported by Witt et al., of low 1,25D in TB [38].

Elevated 1,25D has been found previously in TB patients [31] but in that study, 25D was not measured, so the authors speculated that elevated 1,25D could lead to substrate (25D) depletion, and hence that vitamin D supplementation could be worthwhile. We have now shown that vitamin D substrate (25D), present at concentrations a magnitude higher than 1,25D, is not depleted in TB patients by conversion to 1,25D.

1,25D concentration can be considered a proxy for macrophage activation in this setting. Our finding that higher baseline 1,25D may be advantageous, given its association with ultimate weight gain, could reflect an advantage to the host provided by a robust macrophage immune response at baseline. Also, 1,25D has immunomodulatory properties which may mitigate lung damage, such as inhibition of Th1 cytokines including interferon gamma [8], and suppression of metalloproteinases [39]. An alternative explanation is that a high disease burden at baseline, and therefore high 1,25D levels (Fig. 4a), is associated with greater relative weight gain with treatment; however, this is less likely since baseline weight/BMI were unassociated with 1,25D, and baseline weight was included in the model testing the association between 1,25D and weight gain. The finding that persistent elevation in 1,25D was indicative of a poorer radiological outcome fits with a hypothesis that ongoing macrophage activation in the lung contributes to persisting inflammation and lung damage.

One of our hypotheses was that 25D may behave as a negative acute phase reactant in TB, as has been documented in other conditions [25–28], due to protein-binding and the association between low protein and inflammatory disease states. However, these data do not strongly support this - while 25D was associated with concentrations of binding proteins, VDBP did not differ between TB and controls and did not rise over time, and the spontaneous rise in 25D with treatment, which has been shown previously [22, 40], was slight.

Limitations of this study include that the control subjects were not matched on sex,ethnicity or BMI, but rather, represented members of society who agreed to participate. We sought controls who would resemble the TB patients by including family members, but inevitable differences were evident between the groups. While the multivariable models controlled for these differences, this may still limit conclusions that can be drawn. Missing data occurred for some measures at given time points, chiefly due to patient losses to follow up (Fig. 1); this could result in bias, but the overall effect would have been to diminish power to detect differences between groups and over time. Our endpoints regarding TB outcomes were clinical and radiological rather than more robust microbiological endpoints, due to the homogeneity of microbiological outcomes; also, we were unable to include long-term TB relapse as an outcome. We did not measure UV exposure or diet, hence changes in these may have contributed to the longitudinal increase in total 25D concentration. However, diet in this setting contributes only marginally to vitamin D intake, and all participants were ambulant outpatients at enrolment mostly still engaged in their usual employment, so UV exposure is unlikely to have changed substantially over time. We did not have 25D or 1,25D concentrations prior to onset of TB for obvious reasons; such prospective data would provide clarity on how perturbation in 25D metabolism relates to the onset of active infection. Also, 25D or 1,25D measures were made from peripheral blood samples, not the intracellular compartment, but it is likely that high plasma 1,25D concentration is reflected in high intracelluluar 1,25D bioavailability.

## Conclusions

Our findings show the relationship between 25D and 1,25D in active tuberculosis, and indicate that extra-renal production of 1,25D was high, and was able to occur despite 25D being in the deficient range. Ongoing elevation of 1,25D, a marker of macrophage activation, was associated with worse lung radiological outcomes. 1,25D warrants testing in larger datasets including TB patients less responsive to treatment, such as people with multi-drug resistant TB, to assess its utility as a marker of tuberculosis severity and treatment response.
